# Validation of a Reporter Cell Line for Flavivirus Inhibition Assays

**DOI:** 10.1128/spectrum.05027-22

**Published:** 2023-02-14

**Authors:** Tatiana M. T. Rezende, Gabriella Macera, Leo Heyndrickx, Johan Michiels, Sandra Coppens, Hendrik Jan Thibaut, Kai Dallmeier, Marjan Van Esbroeck, Johan Neyts, Kevin K. Ariën, Koen Bartholomeeusen

**Affiliations:** a Institute of Tropical Medicine, Department of Biomedical Sciences, Virology Unit, Antwerp, Belgium; b KU Leuven Department of Microbiology, Immunology and Transplantation, Rega Institute, Laboratory of Virology and Chemotherapy, Translational Platform Virology and Chemotherapy (TPVC), Leuven, Belgium; c KU Leuven Department of Microbiology, Immunology and Transplantation, Rega Institute, Laboratory of Virology and Chemotherapy, Molecular Vaccinology and Vaccine Discovery (MVVD), Leuven, Belgium; d Institute of Tropical Medicine, Department of Clinical Sciences, Clinical Reference Lab, Antwerp, Belgium; Regional Centre for Biotechnology

**Keywords:** DENV1 to -4, Hec1a-IFNB-Luc, Japanese encephalitis virus, TBEV, USUV, WNV, YFV, ZIKV, flavivirus, luciferase, reporter cell line

## Abstract

Here, we report the validation of a new reporter cell line, Hec1a-IFNB-Luc, for use in inhibition studies of various flaviviruses relevant to human pathology. The reporter system allows the detection of viral replication after luciferase gene activation driven by an interferon beta (IFN-β) promoter. We found the reporter cell line to be highly responsive to all 10 flaviviruses tested, including the 4 dengue virus serotypes. The applicability of the Hec1a-IFNB-Luc reporter cell line for serodiagnostic purposes in neutralizing antibody assays was confirmed by comparison of its sensitivity and specificity to those of “gold-standard,” clinically applied, cytopathic effect-based assays, showing comparable performances. The reporter cell line used for the assessment of viral inhibition by small-molecule antiviral compounds was also confirmed, and the sensitivity of the Hec1a-IFNB-Luc reporter cell line was compared to those from published data reporting on the activity of the antivirals in various other assays, indicating that the Hec1a-IFNB-Luc reporter cell line allowed the determination of the inhibitory capacity at least as sensitive as alternative assays. By measuring luciferase activity as a proxy for viral replication, the reporter cell line allows early detection, reducing the time to results from often 5 to 7 days to 3 days, without the need for optical inspection of cytopathic effects, which often differ between viruses and cell lines, streamlining the development of flavivirus assays.

**IMPORTANCE** The Hec1a-IFNB-Luc reporter cell line allows the detection of all 10 flaviviruses tested, including the 4 dengue virus serotypes. Its use for serodiagnostic purposes, measuring neutralizing antibody activity in sera, and the assessment of the antiviral activities of small-molecule compounds was confirmed, and it was found to be comparable to clinically applied assays. The Hec1a-IFNB-Luc reporter cell line allows the rapid and quantitative determination of antiviral effects on multiple human pathological flaviviruses using a single protocol.

## INTRODUCTION

The genus *Flaviviridae* harbors multiple human pathogens and is notably prevalent among arthropod-borne viruses (arboviruses). Some of the more widespread flaviviruses with epidemic potential are the mosquito-transmitted dengue virus (DENV), yellow fever virus (YFV), and Zika virus (ZIKV) and the tick-transmitted tick-borne encephalitis virus (TBEV). Different flaviviruses often cocirculate, and their comparable acute clinical presentations do not allow differential diagnoses. The correct identification of the causative flavivirus is further complicated by substantial sequence homologies between related flaviviruses, which limits the specificity of various serological assays aiming to identify viral proteins or virus-targeting antibodies, while the use of more specific molecular tests aiming to identify viral genomic material is limited because of the typically short viremic phase (1, 2). Knowledge of the causative agent is, however, important for the informed monitoring of disease progression and potential complications as well as for the gathering of epidemiological data (2).

The cell-based plaque reduction neutralization test (PRNT) is considered the most accurate method for the serological diagnosis of flavivirus infection due to its specificity and sensitivity and is often employed to verify first-line antigen-based rapid test results (3). The PRNT or comparable assays are based on the detection of cytopathic effects (CPE) that the virus of interest elicits in the cell line used. However, even related flaviviruses can present with different CPE signatures after different incubation periods and varying with the specific cell lines and cell culture protocols in use. Moreover, the readout of such CPE-based assays often relies on laborious and subjective visual inspection, potentially limiting consistency, comparability, and throughput.

Flaviviruses are positive single-stranded RNA viruses that, like all RNA viruses, produce double-stranded RNA (dsRNA) intermediates during viral genome replication in the infected cell. These dsRNA species are recognized by cytoplasmic innate immune dsRNA sensors, MDA5 and RIG-I, and the endosomal Toll-like receptor 3 (TLR3) (4). The activation of signaling cascades following the detection of the viral dsRNA converges on the activation of the antiviral interferon beta (IFN-β) gene. To utilize this intrinsic detection mechanism of replicating RNA viruses by the cell as a readout for viral replication, we selected a monoclonal Hec1a endometrial carcinoma cell line stably carrying a luciferase reporter gene under the control of the IFN-β gene promoter. The reporter cell line allows sensitive detection across a broad panel of flaviviruses relevant to human pathology in a uniform and short time frame of 3 days. The luciferase readout allows the convenient and objective quantification of viral replication and its inhibition. We report the validation of the reporter cell line as a specific and sensitive tool for both serodiagnostic neutralization assays for flaviviruses in a direct comparison to PRNTs as performed in a reference laboratory, and antiviral compound testing using previously reported reference compounds as benchmarks.

## RESULTS

### Characterization of the reporter cell line response to flavivirus infection.

To assess the responsiveness of the Hec1a-IFNB-Luc reporter cell line to flavivirus infection, titrations were performed on a representative panel of human-pathogenic flaviviruses. These included yellow fever virus (YFV), two strains of tick-borne encephalitis virus (TBEV) (TBEV strain Hypr [TBEV-Hypr] and TBEV strain Neudörfl [TBEV-Neud]), Zika virus (ZIKV), Japanese encephalitis virus (JEV) (SA14), West Nile virus (WNV), Usutu virus (USUV), and dengue virus serotype 1 (DENV-1) to DENV-4.

**FIG 1:**
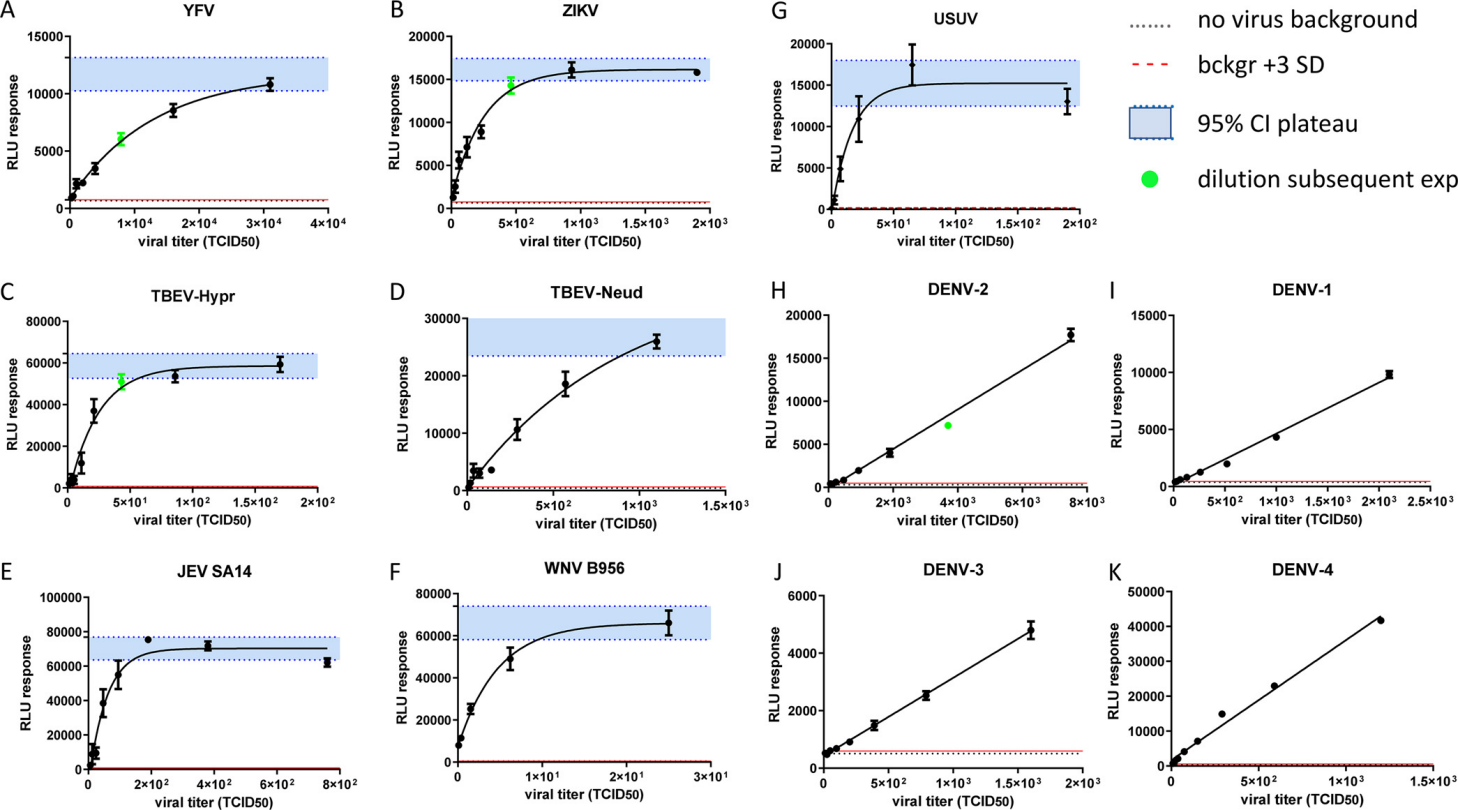
Luciferase activity is stimulated by flavivirus infection of the Hec1a-IFNB-Luc reporter cell line. Hec1a-IFNB-Luc reporter cells were infected for 72 h with titrations of the indicated flavivirus stocks before the assessment of luciferase activity. The TCID_50_ viral titers quantified for each virus stock are listed in Materials and Methods. (A to G) The luciferase activity response to infection with YFV, ZIKV, TBEV-Hypr, TBEV-Neud, JEV SA14, WNV B956, and USUV was fitted with a one-phase exponential association curve using GraphPad. The 95% confidence interval of the plateau phase (95% CI plateau) is indicated in blue. (H to K) The luciferase activity response to infection with DENV-1 was fitted by linear regression using GraphPad. The luciferase activity in the absence of infection is indicated (no virus background). Three times the standard deviation of the background signal is indicated (Bckgr +3 SD). Viral stock dilutions used in subsequent virus inhibition assays are indicated for YFV, ZIKV, TBEV-Hypr, and DENV-2. Replicate titrations are shown (*n* = 6), and standard deviations are indicated.

Measurements of the luciferase activity 72 h after infection showed that each of the 10 tested flaviviruses consistently stimulated the IFN-β promoter-driven reporter gene (Fig. 1). Each virus stimulated luciferase activity to levels significantly above the background signal of uninfected cells (Fig. 1, dotted lines) and the cutoff value of the background ±3 times the SD (standard deviation) (Fig. 1, red lines), a prerequisite for the subsequent use of the reporter system in viral inhibition assays.

The reporter cell line response was fitted by a one-phase exponential association curve for YFV, ZIKV, TBEV, JEV, WNV, and USUV (Fig. 1A to G and Table 1) and showed good reproducibility between independent experiments (see Fig. S1 in the supplemental material). One-phase exponential association fitting allowed the quantitation of the 95% confidence interval (CI) of the plateau phase that follows the exhaustion of the reporter cell response to larger amounts of virus (Fig. 1A to G, blue areas, and Table 1). The absence of a plateau phase for the dengue virus serotypes likely reflects the low infectibility of the cell line and/or nonexhaustive concentrations of the virus stocks, compared to the other flavivirus stocks, and the reporter cell response was fitted by linear regression (Fig. 1H to K and Table 1).

**TABLE 1:** Curve fitting and calculations of the 95% CI of the plateau for the reporter cell line response to titrations of viral stocks^*a*^

Virus	Curve fit type	*R* ^2^	95% CI of the RLU plateau
YFV	One-phase exponential	0.93	10,257–13,153
ZIKV	One-phase exponential	0.88	14,834–17,435
TBEV-Hypr	One-phase exponential	0.87	52,636–64,492
TBEV-Neud	One-phase exponential	0.90	23,459–46,901
JEV SA14	One-phase exponential	0.83	63,636–76,907
WNV B956	One-phase exponential	0.95	58,198–74,055
USUV	One-phase exponential	0.69	12,471–17,991
DENV-1	Linear	0.98	
DENV-2	Linear	0.97	
DENV-3	Linear	0.95	
DENV-4	Linear	0.99	

a RLU, relative light units.

To validate the use of the Hec1a-IFNB-Luc reporter cell line in virus inhibition assays in subsequent experiments, viral stock dilutions of YFV, TBEV-Hypr, ZIKV, and DENV-2 were selected to (i) stimulate luciferase reporter activity significantly above the background, (ii) remain below the plateau phase, and (iii) allow a maximum dynamic range for the quantitation of viral inhibitory activities (Fig. 1A to C and H, dilutions indicated by green dots). A linear response was verified for YFV, ZIKV, TBEV-Hypr, and DENV-2 up to the dilution selected for further experiments (Table 1; Fig. S2). The slopes of the linear regression curve were found to not be significantly different between independent titrations for each virus (Fig. S2).

These data show that the Hec1a-IFNB-Luc reporter cell line has a quantifiable and reproducible response to flavivirus infection that would allow convenient assaying of antiviral treatments.

### Application of the reporter cell line to antiviral compound screening.

To validate the Hec1a-IFNB-Luc reporter cell line as a tool for screening antiviral compounds, we assessed the inhibitory capacities of two adenosine analog RNA-dependent RNA polymerase inhibitors, NITD008 and 7DMA (7-deaza-2’-C-methyladenosine), against DENV-2, ZIKV, TBEV, and YFV. Both compounds have previously been described as broad-spectrum *in vitro* flavivirus inhibitors (5–12).

**FIG 2:**
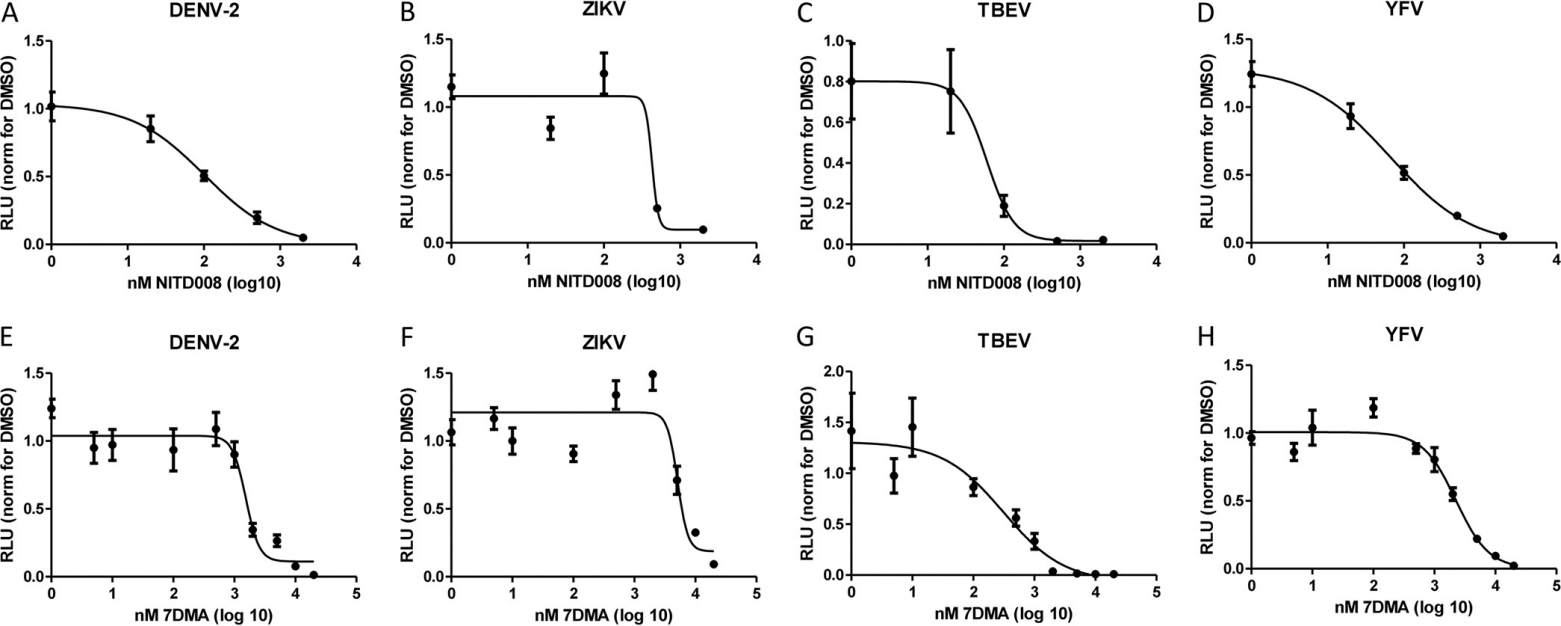
Quantification of the antiviral activity of the flavivirus inhibitors NITD008 and 7DMA using the Hec1a-IFNB-Luc reporter cell line. Hec1a-IFNB-Luc reporter cells were coincubated with the indicated flaviviruses and dilutions of antiviral compounds for 72 h. Luciferase activities were normalized to the corresponding amounts of DMSO. DENV-2, ZIKV, TBEV, and YFV were incubated with serial dilutions of NITD008 (A to D) or 7DMA (E to H). Inhibition curves were fitted with a four-parameter variable-slope curve using GraphPad. Replicate titrations are shown (*n* = 6), and standard deviations are indicated.

Viral stock dilutions of DENV-2, ZIKV, TBEV, and YFV, selected as described above (Fig. 1, green dots), were coadministered to the reporter cells with titrations of the compounds. Dimethyl sulfoxide (DMSO) controls were performed in parallel and served to normalize the luciferase reporter activity (Fig. S3). Increasing amounts of both compounds reduced the luciferase reporter activity by the four separate flaviviruses, and the inhibition profiles were readily fitted with a four-parameter variable-slope curve (Fig. 2A to H).

**FIG 3:**
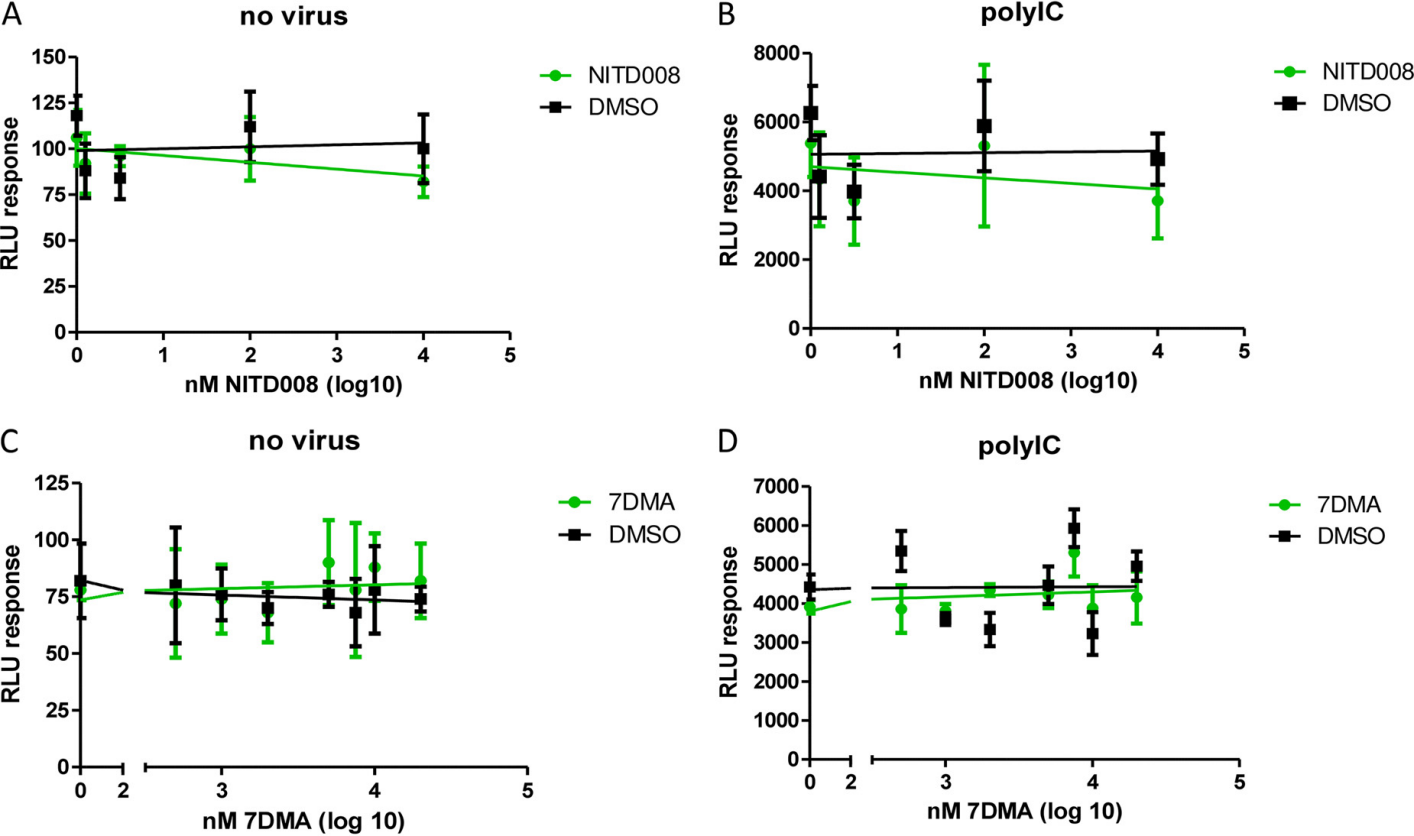
NITD008 or 7DMA treatment does not affect the background or nonviral stimulation of luciferase activity by the Hec1a-IFNB-Luc reporter cell line. Hec1a-IFNB-Luc reporter cells were treated with serial dilutions of NITD008 or 7DMA alone for 72 h (A and C) or simultaneously with the transfection of poly(I·C) for 24 h (B and D). In parallel, the corresponding dilutions of DMSO were assessed. The data were fitted with linear regression using GraphPad. Replicate titrations are shown (*n* = 6), and standard deviations are indicated.

To verify that the reporter cell line allowed the assessment of viral inhibition by the compounds rather than the suppression of innate immune sensing of viral replication, IFN-β gene activation, or outright inhibition of luciferase activity, through either specific effects or cellular toxicity, we determined the effects of NITD008 and 7DMA on the background luciferase activity and nonviral stimulation of the IFN-β promoter-driven reporter luciferase. The titration of NITD008 and 7DMA in noninfected Hec1a-IFNB-Luc reporter cells did not affect the background luciferase activity (Fig. 3A and C), indicating that the compounds did not impact basal luciferase expression or activity. Importantly, the viral replication-independent stimulation of luciferase activity by the transfection of poly(I·C), a double-stranded RNA mimic, was similarly unaffected by NITD008 or 7DMA (Fig. 3B and D), indicating that the decrease in reporter activity upon DENV-2, ZIKV, TBEV, and YFV infection in the presence of the antiviral compounds was due to the inhibition of viral replication and further supporting the use of the reporter cell line as a robust tool for the characterization of antiflaviviral compounds.

The half-maximum inhibitory concentrations (IC_50_s) of NITD008 and 7DMA for each virus were derived from three biological repeat experiments and compared to previously reported IC_50_ values derived from various other virological assays (Table 2). Overall, we found that the Hec1a-IFNB-Luc reporter cell line allowed the determination of the inhibitory capacity of antiviral compounds at least as sensitive as other methods. Specifically, the IC_50_ values determined using the Hec1a-IFNB-Luc reporter cell line for NITD008 and 7DMA against ZIKV were within the range of previously reported IC_50_ values, while the IC_50_ values for both compounds against DENV-2, YFV, and TBEV were on average lower than previously reported IC_50_s using different methods (Table 2, compare the IC_50_s in the literature to the IC_50_s in this study). Several variables may explain this generally somewhat higher antiviral activity observed in our reporter cells, such as the use of potentially smaller viral doses than those in the other assays, virus strain-dependent variables, or cell type-specific differences in the uptake or activation of the nucleoside analog prodrugs.

**TABLE 2:** Comparison of IC_50_ values determined previously using various methods and those determined here for NITD008 and 7DMA against DENV-2, ZIKV, TBEV, and YFV^*a*^

Compound and virus	Method	Reference	IC_50_ (nM) in the literature	Mean IC_50_ (nM) ± SD in this study
NITD008				
DENV-2	PRA	11	0.64	0.158 ± 0.082
	PRA	9	4.2	
	LRV	22	0.64	
ZIKV	PRA	23	0.137–0.24	0.323 ± 0.147
	RT-qPCR	23	0.283–0.95	
	LRV	22	0.4	
TBEV	CPE	8	0.9 ± 0.29	0.046 ± 0.019
	IHC	8	0.226	
	VTR CPE	8	2.99	
YFV	LRV	22	0.32	0.094 ± 0.039
7DMA				
DENV-2	CPE	10	15	2.113 ± 0.680
	RT-qPCR	7	14 ± 1.2	
	HCI	7	6.7 ± 2.2	
ZIKV	CPE	12	20 ± 15	4.854 ± 0.466
	VTR CPE	12	9.6 ± 2.2	
	PRA	12	1.3	
	IHC	12	5.7 ± 2.2	
	CPE	5	8.92 ± 3.32	
TBEV	PRA	6	5.1 ± 0.4	0.348 ± 0.074
	CPE	24	1.07 ± 0.03	
YFV	CPE	10	15	3.121 ± 0.786

a PRA, plaque reduction assay; LRV, luciferase reporter virus; RT-qPCR, reverse transcription-quantitative PCR to detect viral RNA in the infected cell; CPE, cytopathic effect readout during drug treatment; IHC, immunohistochemistry; VTR CPE, virus reduction in the supernatant by a CPE assay; HCI, high-content imaging of a fluorescent reporter virus.

Together, these findings establish the Hec1a-IFNB-Luc reporter cell line as a robust and sensitive tool to characterize and assess the antiviral potency of candidate therapeutics and suggest its applicability for the high-throughput screening of compound libraries.

### Application of the reporter cell line for serodiagnosis.

The determination of neutralizing antibody titers in serum against a suspected virus is a commonly used diagnostic method to establish previous or ongoing infection. The plaque reduction neutralization test (PRNT) is considered the gold standard in serodiagnosis due to its sensitivity and specificity.

**FIG 4:**
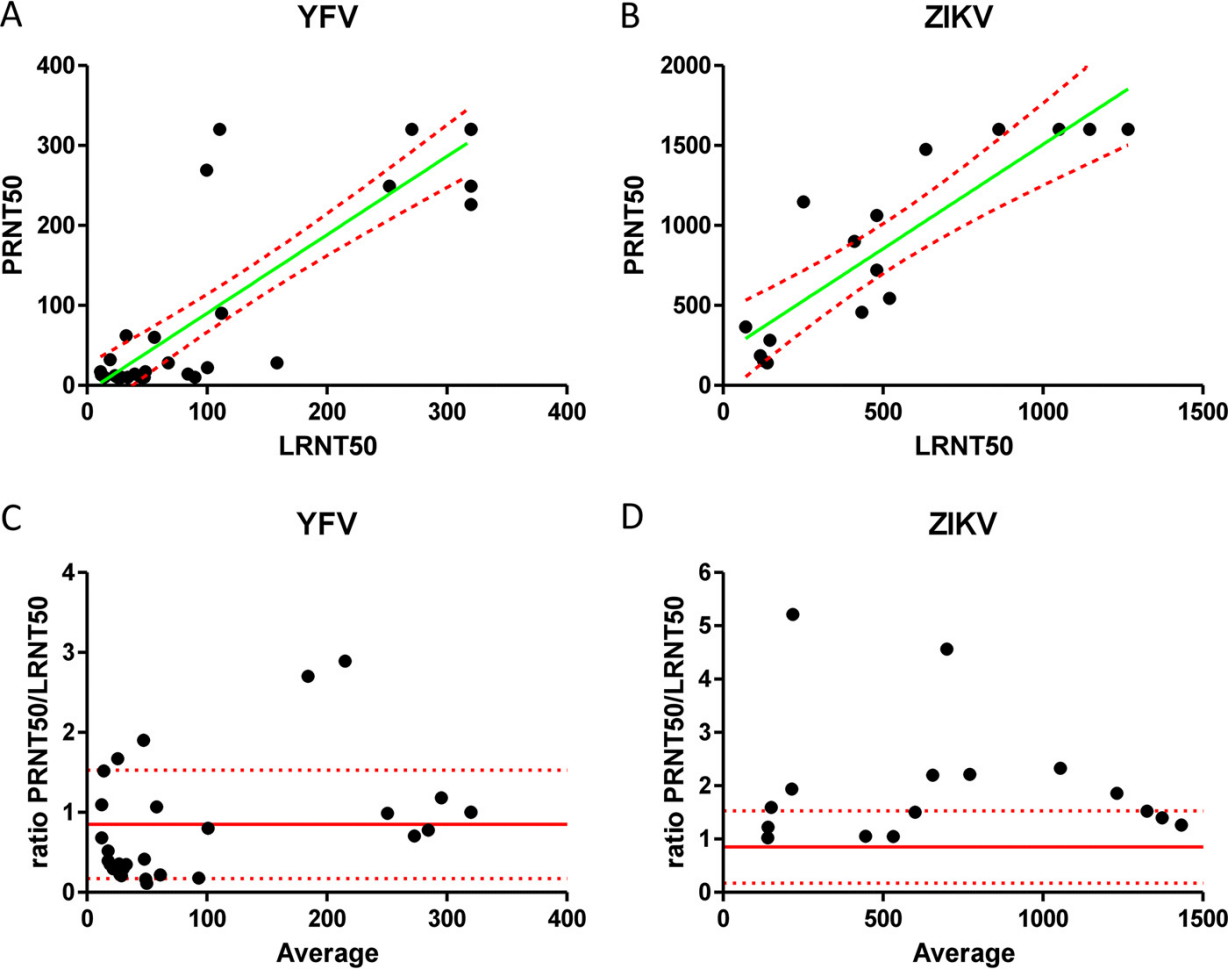
Correlation of half-inhibitory concentrations of neutralizing sera using a PRNT and the Hec1a-IFNB-Luc reporter cell line. Neutralizing antibody titers were determined using the Hec1a-IFNB-Luc reporter cell line for sera that were previously measured using a PRNT. (A and B) Serial dilutions of sera with suspected neutralizing activity against YFV (A) and ZIKV (B) were incubated with the corresponding viruses, and inhibition curves were fitted with a four-parameter variable slope to determine the half-inhibitory concentration (LRNT_50_) (*x* axis). LRNT_50_ values were compared to half-inhibitory concentrations determined by a PRNT (PRNT_50_) (*y* axis). LRNT_50_ values were capped to maximum values corresponding to maximum PRNT_50_ values (320 for YFV, 1,600 for ZIKV, and 320 for TBEV). Six replicate titrations were performed for each serum sample to determine the LRNT_50_ and PRNT_50_. Linear regression was performed using GraphPad, and the *R*^2^ values were calculated (green lines, linear regression; red dotted lines, 95% CI). (C and D) The same data were used to produce Bland-Altman plots of the PRNT_50_/LRNT_50_ ratios for YFV (C) and ZIKV (D) to assess the propensity of either assay to report increased or decreased relative values across the range of values (red lines, bias; red dotted lines, SD of bias).

To support the application of the Hec1a-IFNB-Luc reporter cell line in serodiagnostic assays, we compared its performance to that of the PRNT for sera containing neutralizing antibodies against YFV or ZIKV (Fig. 4). The sera used were obtained from patients suspected of having ZIKV infection or from nonhuman primates (NHPs) vaccinated against YFV that had previously been assessed by the Arbovirus National Reference Center at the Institute of Tropical Medicine (ITM) using a PRNT (13). Viruses were incubated with serum dilutions before addition to the reporter cell line, luciferase activity was measured after 72 h, and inhibition profiles were fitted with a four-parameter variable-slope curve. Half-inhibitory concentrations were calculated and are referred to as LRNT_50_ (50% luciferase reduction neutralizing titer) values here.

For the PRNT, half-inhibitory concentration (PRNT_50_) values were determined using the Reed-Muench method (14), capping the maximum PRNT_50_ values at the highest dilution values assayed in the experiments. LRNT_50_ values were similarly capped at the corresponding values to allow comparisons (PRNT_50_ for YFV of >320 and PRNT_50_ for ZIKV of >1,600). Comparison of the LRNT_50_ and PRNT_50_ values showed a good correlation between the two methods for YFV and ZIKV (Fig. 4A and B) (*R*^2^ = 0.78 and 0.76, respectively). Comparison of the relative deviations between the two assays across the range of values showed no propensity for reporting disparate values in either the higher or the lower range (Fig. 4C and D).

**FIG 5:**
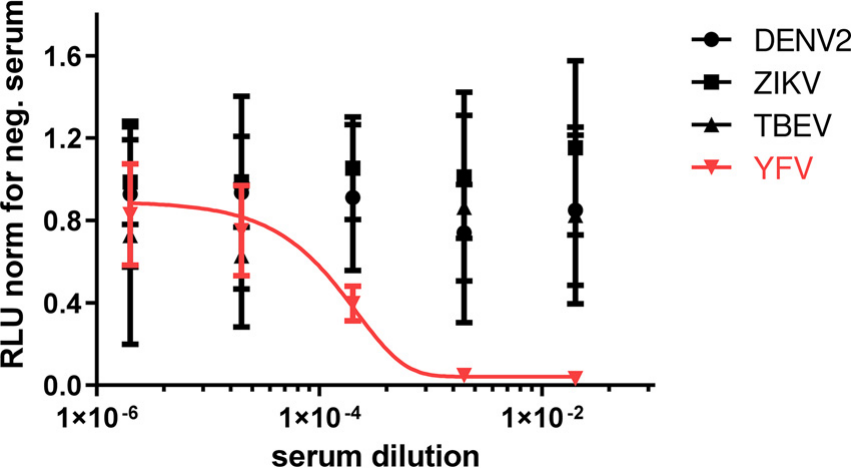
The Hec1a-IFNB-Luc reporter cell line allows the assessment of the specific serum neutralizing capacity. YFV-neutralizing serum was assessed for neutralizing activity toward DENV-2, ZIKV, TBEV, and YFV. Nonneutralizing serum was assayed in parallel for each virus and served to normalize the luciferase activity. Replicate titrations are shown (*n* = 6), and standard deviations are indicated.

Cell-based assays such as the PRNT allow increased specificity for the detection of virus-targeting antibodies compared to other serodiagnostic assays such as enzyme-linked immunosorbent assay (ELISA)-based methods. To assess whether the use of the Hec1a-IFNB-Luc reporter cell line allowed similar specificity, we compared the reactivities of sera from nonhuman primates vaccinated with a live-attenuated YFV vaccine toward YFV, TBEV, ZIKV, and DENV-2. As generally described for the PRNT, the reporter cell line allowed the detection of YFV-neutralizing activity without registering cross-reactivity toward ZIKV, TBEV, and DENV-2 (Fig. 5).

Together, these data show that the Hec1a-IFNB-Luc reporter cell line allows the assessment of flavivirus-neutralizing antibody titers with sensitivity and specificity comparable to those of the gold-standard CPE-based PRNT.

## DISCUSSION

Cell-based assays are used for the assessment of viral replication, the preclinical evaluation of the inhibitory activity of antiviral compounds, or the determination of the presence of neutralizing antibodies in patient serum for serodiagnostic purposes.

The plaque reduction neutralization test (PRNT) is a method commonly used for the determination of neutralizing antibody activity in sera from patients to assess ongoing or previous infection with a suspected virus for diagnostic purposes or to assess vaccine-induced humoral responses. The PRNT is considered the gold standard in serodiagnosis as it provides a sensitive and specific readout compared to, for example, non-cell-based methods such as ELISAs, indirect immunofluorescent-antibody tests (IFATs), and rapid diagnostic tests (RDTs). Flaviviruses constitute a major portion of human-pathogenic arthropod-borne viruses worldwide, and different flaviviruses as well as related serotypes (e.g., DENV-1 to -4) cocirculate in many regions (such as DENV, ZIKV, and YFV in Latin America [15–17]; DENV, ZIKV, and JEV in Asia [18]; DENV, WNV, and YFV in Central Africa [19]; and DENV and ZIKV in West Africa [20]), underscoring the need for optimal specificity for flavivirus serodiagnosis. However, analysis by PRNTs is time-consuming and requires labor-intensive visual readout of cytopathic effects (CPE), and the convenient assessment of separate flaviviruses can be hampered by differences in viral replication dynamics or CPE in a given cell line.

We report the validation of a reporter cell line that allows the detection of a broad spectrum of flaviviruses to assess the inhibitory activity of antiviral molecules and neutralizing antibodies in serum.

During genomic replication, flaviviruses produce dsRNA intermediates, which are recognized by cytosolic (RIG-I and MAD5) and endosomal (TLR3) pattern recognition receptors (PRRs) (4). The activation of these PRRs ultimately activates IFN-β gene expression. The Hec1a cell line was modified to carry a stably integrated luciferase reporter gene under the control of an IFN-β gene promoter.

We found that luciferase reporter gene expression was consistently and potently activated by infection of the reporter cell line by all flaviviruses tested. The selected flaviviruses represent important human-pathogenic arboviruses and included two variants of TBEV and the four DENV serotypes. This suggests that the Hec1a-IFNB-Luc reporter cell line represents a convenient tool to rapidly assess different circulating strains, lineages, and variants without the need to identify a cell line that presents CPE or that would require the time-consuming production of molecular reporter clones of newly emerging viruses to allow their detection.

Using a given cell line for PRNTs, the time before CPE emerge can vary substantially among flaviviruses, and this routinely requires at least a 5-day incubation before accurate visual determination can occur. In addition, some flaviviruses do not present with overt CPE, which can hamper quantification. As the Hec1a-IFNB-Luc reporter cell line detects viral dsRNA production at the early stages of viral replication, there is no need for the development of CPE, and viral infection was reliably detected for all viruses tested after a 3-day incubation, which could conceivably be shortened for select more rapidly replicating flaviviruses such as YFV, ZIKV, or TBEV.

Importantly, we found that the sensitivity and specificity of the Hec1a-IFNB-Luc reporter cell line were comparable to those of the PRNT performed on clinical serum samples from patients and vaccine-treated NHPs analyzed by the national Arbovirus Reference Lab at the Institute of Tropical Medicine.

Similarly, we found a good correlation with the previously reported activities of two broadly studied antiviral compounds. This benchmarking establishes the Hec1a-IFNB-Luc reporter cell line as a reliable and convenient alternative to other cell-based assays, allowing shorter incubation times and a quantitative luciferase readout, obviating the visual assessment of CPE.

Pilot studies further indicated that the reactivity of the cell line was stable over the course of several months in culture, and sensitive readouts were retained using up to 10-times-fewer cells (data not shown), suggesting easy adaptability to high-throughput and (semi)automated formats.

## MATERIALS AND METHODS

### Derivation, maintenance, and use of the Hec1a-IFNB-Luc reporter cell line.

Hec1a cells (21) (obtained from the ATCC [ATCC HTB-112]) were previously shown to allow infection by different arboviruses and to support innate immune sensing and signaling, converging on IFN-β gene activation, in our laboratory. Hec1a cells were transiently transfected with a plasmid containing the luciferase reporter gene driven by the IFN-β promoter and carrying a hygromycin resistance expression cassette. Polyclonal cell populations were selected in hygromycin-containing medium. Monoclonal cell lines were derived by limiting dilution. Forty monoclonal cell lines were subsequently assessed for luciferase activation responses to transient poly(I·C) transfection. One monoclonal cell line was selected based on its minimal background luciferase activity and its highest responsiveness to stimulation with poly(I·C).

Cells were grown in McCoy’s 5A medium containing 10% fetal bovine serum (FBS) (Gibco), l-glutamine (Sigma), and 5% hygromycin (50 mg/mL) (Invitrogen) and incubated at 37°C with 7% CO_2_.

For luciferase response measurements, 20,000 cells were plated onto a 96-well plate 24 h before treatment. After treatment-specific incubation, luciferase activity was measured using the Steadylite plus reporter gene assay system (PerkinElmer) according to the manufacturer’s instructions. The cell lysate was transferred to white OptiPlates (PerkinElmer), and luminescence was measured using a luminometer (Berthold). The luminescence signal was measured for 1 s per well.

One microgram of poly(I·C) (Sigma-Aldrich) was transfected using Fugene 6 (Promega) according to the manufacturer’s instructions. Cells were incubated in McCoy’s 5A medium containing 2% FBS (Gibco) and l-glutamine (Sigma), and reporter luciferase activity was measured after 24 h.

Virus dilutions, in the presence or absence of neutralizing serum or an antiviral compound, were added to the cells in 150 μL McCoy’s 5A medium containing 2% FBS (Gibco) and l-glutamine (Sigma), and reporter luciferase activity was measured after 72 h. Antiviral compounds were suspended in DMSO.

Serum neutralization assays were performed by the preincubation of serum and the virus suspension in 150 μL McCoy’s 5A medium containing 2% FBS (Gibco) and l-glutamine (Sigma) at 37°C with 7% CO_2_ for 1 h before addition to the reporter cells. Each serum dilution was assayed in 6 replicates, and each plate contained 6 replicates of a negative control, virus in the presence of 10% pooled human serum (PHS) (previously determined to not possess neutralizing activity against the viruses tested) or 2% FBS.

### Viruses and compounds.

NITD008 (catalog number SML2409) and 7DMA (catalog number SML3200) were obtained from Sigma-Aldrich. TBEV-Hypr was a kind gift from Hana Zélena, Public Health Institute, Ostrava, Czech Republic. TBEV-Neudörfl was a kind gift from the Medical University of Vienna, Austria. WNV (B956) was obtained through BEI Resources, NIAID, NIH (catalog number NR-72). USUV was a kind gift from Mutien Garigliany at the Université de Liège, Belgium. DENV-1 to -4 were obtained through the Belgian National Reference Centre for Arboviruses. DENV-1 was isolated from the serum of a patient from Indonesia, and DENV-2 to -4 were isolated from the sera of patients from Thailand. Virus was propagated on Vero cells, and DENV serotypes were confirmed by serotype-specific PCR. YFV (17D) was a kind gift from the Robert Koch Institute, Berlin, Germany. ZIKV (MR 766) and JEV (SA14) were kind gifts from the Bernhard-Nocht-Institut für Tropenmedizin, Hamburg, Germany. All viruses were propagated in Vero cells, and aliquots were stored at −80°C before use. Viral titers were determined by CPE assays in Vero cells for all viruses except for the titers of TBEV, which were determined in A549 cells. The 50% tissue culture infective doses (TCID_50_) per milliliter of the virus stocks were as follows: 7.9E+06 for WNV, 7.9E+06 for USUV, 4.2E+05 for DENV-1, 1.5E+06 for DENV-2, 1.6E+05 for DENV-3, 4.7E+05 for DENV-4, 1.6E+08 for YFV, 9.3E+07 for ZIKV, 7.6E+06 for JEV, 8.6E+06 for TBEV-Hypr, and 1.1E+07 for TBEV-Neud.

### Sera.

Monkey sera were collected from rhesus macaques vaccinated with the licensed YF17D vaccine Stamaril (13).

To inactivate complement, all sera were heat inactivated by incubation at 56°C for 30 min prior to use.

### Ethics statements.

Monkey sera were sampled from purpose-bred rhesus macaques (Macaca mulatta) housed at the Biomedical Primate Research Centre (BPRC) (Rijswijk, The Netherlands) and vaccinated with Stamaril (Sanofi-Pasteur) upon positive advice by the independent ethics committee (DEC-BPRC) under project license DEC753C issued by the Central Committee for Animal Experiments, according to Dutch law. Approval for the use of patient-derived sera provided by the Arbovirus Reference Centre at the ITM for the validation of the reporter cell line assay for ZIKV neutralization assays was obtained from the ITM Institutional Review Board (reference number 1643/22) and the ethics committee of the University Hospital of Antwerp (EC UZA ID 5041).
