# Supplementary material for: Validation of a Reporter Cell Line for Flavivirus Inhibition Assays
Source: Microbiol Spectr. 2023 Feb 14;11(2):e05027-22. doi: 10.1128/spectrum.05027-22 (PMC10100686; doi:10.1128/spectrum.05027-22)
Supplement: Supplemental file 1 — Supplemental material. Download spectrum.05027-22-s0001.pdf, PDF file, 0.4 MB [file spectrum.05027-22-s0001.pdf]

Suppl Fig.1 **Comparison of repeat titrations**

Repeat titrations (n=6) of indicated flaviviruses were repeated (n=2 or n=3) at least 3 months apart. A-F) luciferase activity responses to infection with YFV, ZIKV, TBEV-Hypr, TBEV-Neudörfl, JEV SA14 and WNV B956 were fitted with a one-phase exponential association curve using graphpad. The plateau phase 95% confidence interval (95% CI plateau) were calculated. Repeat experiments were normalized by the calculated plateau G-J) luciferase activity responses to infection with DENV-1 to DENV-4 were fitted by linear regression and repeat experiments normalized to each other using the highest values.

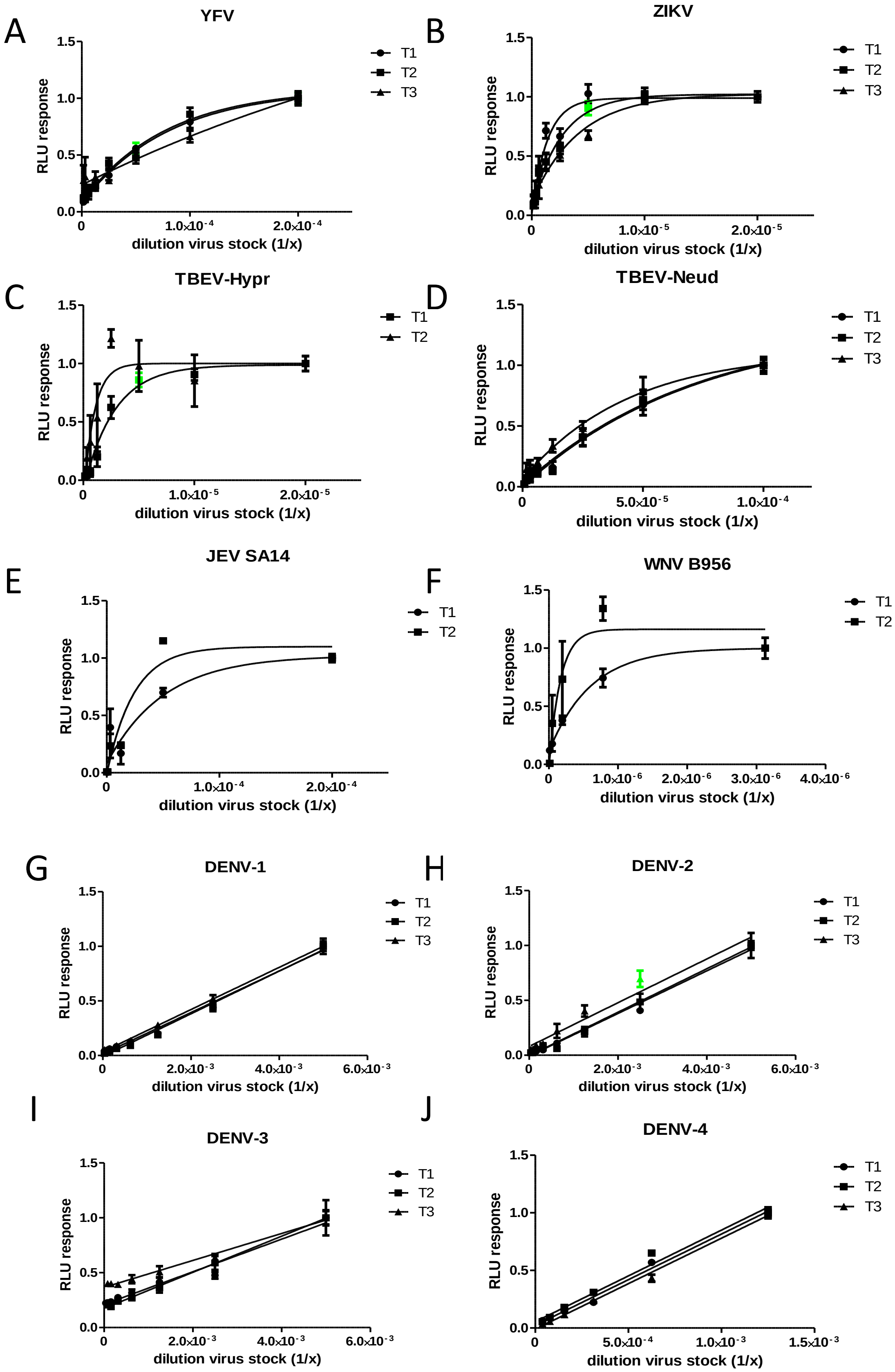

A
YFV
RLU response
dilution virus stock (1/x)
T1
T2
T3
B
ZIKV
RLU response
dilution virus stock (1/x)
T1
T2
T3
C
TBEV-Hypr
RLU response
dilution virus stock (1/x)
T1
T2
D
TBEV-Neud
RLU response
dilution virus stock (1/x)
T1
T2
T3
E
JEV SA14
RLU response
dilution virus stock (1/x)
T1
T2
F
WNV B956
RLU response
dilution virus stock (1/x)
T1
T2
G
DENV-1
RLU response
dilution virus stock (1/x)
T1
T2
T3
H
DENV-2
RLU response
dilution virus stock (1/x)
T1
T2
T3
I
DENV-3
RLU response
dilution virus stock (1/x)
T1
T2
T3
J
DENV-4
RLU response
dilution virus stock (1/x)
T1
T2
T3

Suppl Fig.2 **Slope of linear range is not significantly different between repeat experiments**

Linearity of the range preceding the plateau phase, up to the chosen dilution for further experimentation, was verified for YFV, ZIKV and TBEV by linear regression using graphpad and the slope calculated. Slopes were compared and found not to be significantly different between repeat titrations ($p > 0,05$). P-values are indicated.

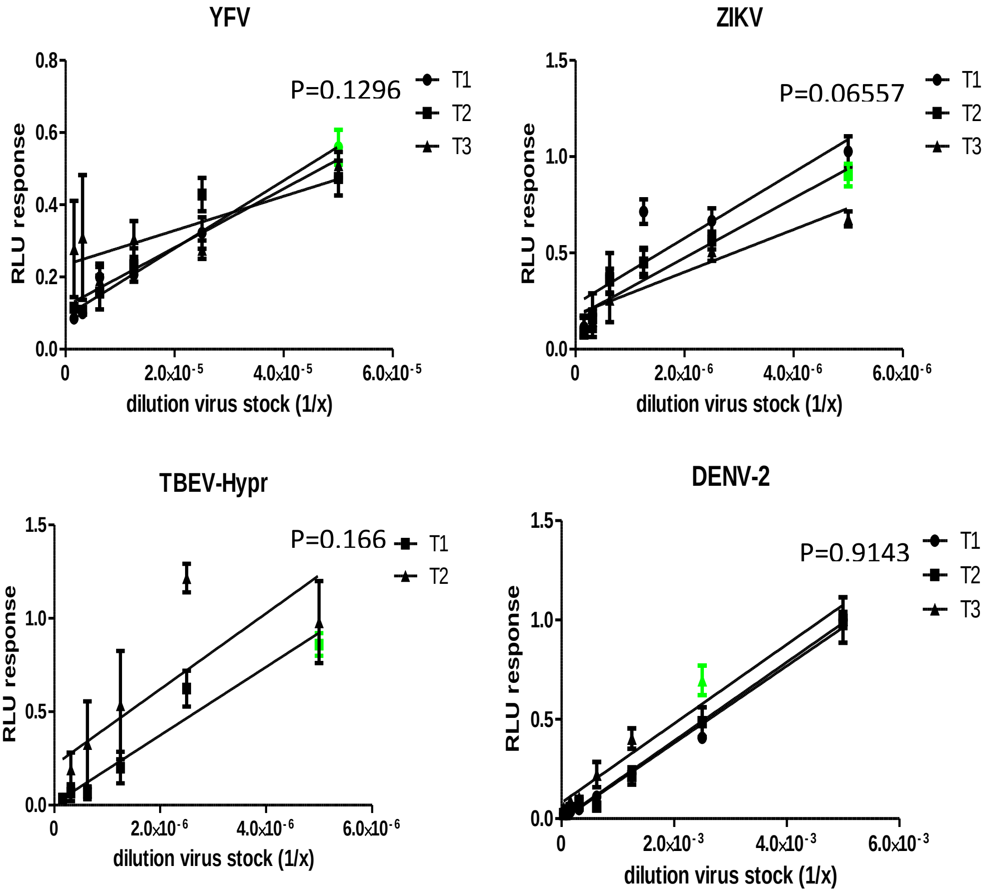
YFV
P=0.1296
T1
T2
T3
RLU response
0.0
0.2
0.4
0.6
0.8
0
2.0x10-5
4.0x10-5
6.0x10-5
dilution virus stock (1/x)
ZIKV
P=0.06557
T1
T2
T3
RLU response
0.0
0.5
1.0
1.5
0
2.0x10-6
4.0x10-6
6.0x10-6
dilution virus stock (1/x)
TBEV-Hypr
P=0.166
T1
T2
RLU response
0.0
0.5
1.0
1.5
0
2.0x10-6
4.0x10-6
6.0x10-6
dilution virus stock (1/x)
DENV-2
P=0.9143
T1
T2
T3
RLU response
0.0
0.5
1.0
1.5
0
2.0x10-3
4.0x10-3
6.0x10-3
dilution virus stock (1/x)

Suppl Fig.3 **Separate titrations of DMSO solubilized antiviral compounds and DMSO alone**

Hec1a-IFNB-Luc reporter cells were co-incubated with indicated flaviviruses and dilutions of antiviral compounds solubilized in DMSO or DMSO alone for normalization purposes. Luciferase activities were measured for DENV-2, ZIKV, TBEV and YFV in presence of increasing amounts of 7DMA (A-D) and equivalent amounts of DMSO only (E-H, “eq DMSO”) or NITD008 (I-L) and equivalent amounts of DMSO alone (M-P, “eq DMSO”) Replicate titrations are shown (n=6) and standard deviations indicated.

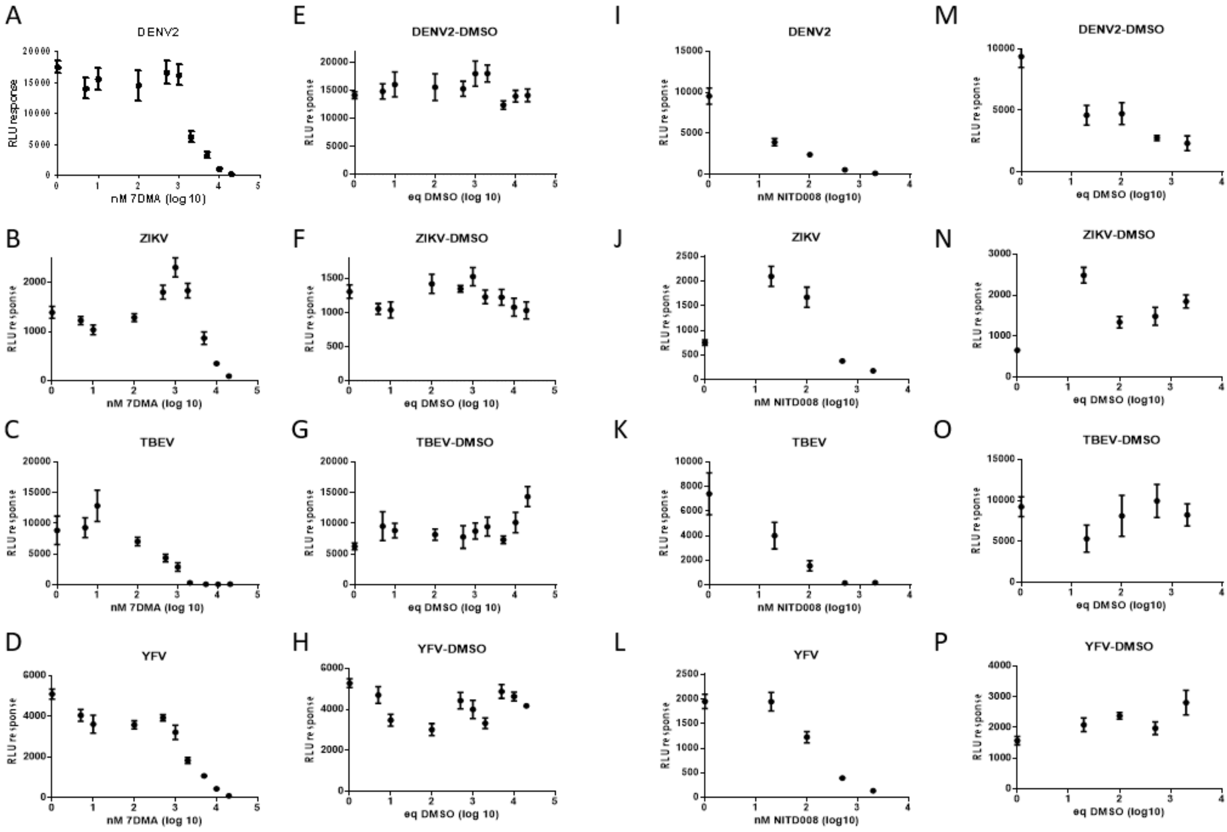
A
DENV2
RLU response
nM 7DMA (log10)
B
ZIKV
RLU response
nM 7DMA (log 10)
C
TBEV
RLU response
nM 7DMA (log 10)
D
YFV
RLU response
nM 7DMA (log 10)
E
DENV2-DMSO
RLU response
eq DMSO (log 10)
F
ZIKV-DMSO
RLU response
eq DMSO (log 10)
G
TBEV-DMSO
RLU response
eq DMSO (log 10)
H
YFV-DMSO
RLU response
eq DMSO (log 10)
I
DENV2
RLU response
nM NITD008 (log10)
J
ZIKV
RLU response
nM NITD008 (log10)
K
TBEV
RLU response
nM NITD008 (log10)
L
YFV
RLU response
nM NITD008 (log10)
M
DENV2-DMSO
RLU response
eq DMSO (log10)
N
ZIKV-DMSO
RLU response
eq DMSO (log10)
O
TBEV-DMSO
RLU response
eq DMSO (log10)
P
YFV-DMSO
RLU response
eq DMSO (log10)
